# Avapritinib in the treatment of systemic mastocytosis with associated acute myeloid leukemia after poor graft function following allogeneic hematopoietic stem cell transplantation: a case study and review of the literature

**DOI:** 10.3389/fonc.2025.1728830

**Published:** 2025-12-15

**Authors:** Ruihua Mi, Xiang Li, Lin Chen, Lin Wang, Yixuan Ma, Yuewen Fu, Xudong Wei

**Affiliations:** The Affiliated Cancer Hospital of Zhengzhou University & Henan Cancer Hospital, Zhengzhou, China

**Keywords:** acute myeloid leukemia, Allo-HSCT, avapritinib, case report, systemic mastocytosis

## Abstract

**Introduction:**

This case is reported due to its novelty in demonstrating the efficacy of avapritinib, a selective *KIT* inhibitor, in a rare systemic mastocytosis-associated acute myeloid leukemia (SM-AML) patient with non-D816V *KIT* mutations and *RUNX1::RUNX1T1* fusion. Avapritinib is established for *KIT* D816V-mutant advanced systemic mastocytosis (AdvSM). However, its role in non-D816V *KIT* mutant SM-AHN post-allogeneic hematopoietic stem cell transplantation (Allo-HSCT) remains unexplored, highlighting the need to document this therapeutic challenge and outcome.

**Case presentation:**

A 15-year-old Asian male with SM-AML underwent induction chemotherapy, targeted therapy, and Allo-HSCT, but experienced graft failure with persistent mastocytosis. Post-Allo-HSCT avapritinib was initiated due to high proportion of mast cells (MCs). After 14 months, the patient achieved minimal residual disease (MRD)-negative complete remission, full donor chimerism (100%), and sustained hematologic recovery without adverse events. MC burden declined from 14.06% to 0.3%, and *RUNX1::RUNX1T1* fusion became undetectable.

**Conclusion:**

This case highlights avapritinib’s potential as a salvage therapy for non-D816V *KIT* mutant SM-AML post-Allo-HSCT, effectively reducing MC clones and restoring donor chimerism. It suggests that avapritinib may bridge therapeutic gaps for atypical *KIT*-mutant systemic mastocytosis with associated hematologic neoplasm (SM-AHN) that is ineligible for Allo-HSCT or relapsed. Prospective trials are warranted to validate its efficacy, optimize dosing, and explore synergies with Allo-HSCT, offering new strategies for these high-risk patients.

## Introduction

Systemic mastocytosis (SM) is a rare clinical heterogeneous disease that is characterized by the clonal proliferation of mast cells (MCs) in extracutaneous organs. *KIT* gene mutations are detected in more than 90% of SM patients, with *KIT* D816V being the most common (1). The prognosis of patients with systemic mastocytosis with associated hematologic neoplasm (SM-AHN) is poor, and the 3-year overall survival (OS) after receiving allogeneic hematopoietic stem cell transplantation (Allo-HSCT) is 74% (2). Furthermore, previous studies have demonstrated significant efficacy of TKIs in treating SM-AHN. However, no reports currently exist regarding TKI therapy for related diseases following failed stem cell transplantation (3). Herein, we report the case of a SM-AML patient with a non-D816V *KIT* mutation who received avapritinib monotherapy after Allo-HSCT failure. The patient achieved minimal residual disease (MRD)-negative complete remission (CR) and was in sustained remission.

## Case presentation

A 15-year-old Asian male patient came to our hospital in July 2021, with a fever of 38.9°C. Past medical, family, and psychosocial history were unremarkable. The routine blood test results were as follows: white blood cell (WBC) count, 9.2×10^9^/L; neutrophil (#N) count, 4.1×10^9^/L; hemoglobin (HGB), 76 g/L; and platelet (PLT) count, 10×10^9^/L. In the peripheral blood smear analysis, 46% of the cells were myeloblasts. The bone marrow smear analysis results were as follows: 1. active hyperplasia, with myeloblasts accounting for 10% of cells; and 2. MCs accounted for 33.6% of cells. The bone marrow biopsy results revealed the following: 1. active proliferation of nucleated cells in bone marrow (approximately 70% of the hematopoietic area) with dense aggregation; 2. granulocytes scattered in the form of immature cells; myeloblasts (approximately 8%), with large cell body, small cytoplasm, and oval or slightly irregular nuclei; and 3. extensive proliferation of MCs (approximately 70%), with some MCs being immature. The immunohistochemistry (IHC) results were as follows: CD34 (+) in small blood vessels, scattered round nucleus cells (+), multiple CD117 (+), multiple MPO (+), CD117+, MCT+, MCC+ in most MCs and CD25+ in some MCs, CD30-, and CD2-. Multiparameter flow cytometry (MFC) showed that abnormal myeloblasts accounted for 11.63% of cells, with CD34, CD117, HLA-DR, CD33, and CD38 expression, weak CD13, CD123, and CD19 expression, in addition, MCs accounted for 4.01% of cells. Karyotype analysis showed 45,X,-Y,t(8,21)(q22;q22)[9]/46,XY[1]. The following gene mutations were identified using next-generation sequencing (NGS): *KIT* (p.Arg420Thr, mutation frequency 18.92%), *KIT* (p. Tyr418_Asp419delinsAla, mutation frequency 19.01%), *KIT* (p. Tyr503_Phe504insLeuArg, mutation frequency 5.92%), and *FAT1* (pArg3729Gly, mutation frequency 49.12%). The *RUNX1::RUNX1T1* fusion gene was detected. The final diagnosis was SM-AHN associated with AML carrying the *RUNX1::RUNX1T1* fusion.

The IA (idarubicin combine with cytarabine) regimen was started in July 2021: Idarubicin (IDA) 20 mg intravenous for 3 days, cytarabine (Ara-C) 200 mg intro venous for 7 days, combined with imatinib mesylate 400 mg po qd. Bone marrow smear analysis in August 2021 showed that myeloblasts accounted for 2% of cells and MCs accounted for 53.2%. MFC-MRD (using multiparameter flow cytometry) deleted 2.36% of abnormal myeloblasts. Fusion gene detection (MOL-MRD) (using quantitative polymerase chain reaction) showed *RUNX1::RUNX1T1/ABL* = 3.40%. In September, the bone marrow smear analysis detected 2% of myeloblasts and 36.0% of MCs, with MFC-MRD detected 0.70% of abnormal myeloblasts. The patient was given a high-dose Ara-C (HDAC) regimen (Ara-C 3.0 g per 12h, d1, 3, 5) combined with dasatinib 50 mg oral daily for 8 days as consolidation treatment. On October 6, dasatinib was discontinued due to unsatisfactory hemogram parameters, considered a potential dasatinib toxicity reaction. The bone marrow smear analysis detected 33.0% of MCs, with MFC-MRD identifying 15.52% of MCs. In October 2201, the patient received the HDAC regimen again. In November 2021, the patient was administered “decitabine 10 mg d1~5” as a bridging therapy, followed by the improved Bu/Cy regimen: Me-CCNU 450 mg -6d, Ara-C 3.0 g q12h -6~-5d, Bu 45 mg q6h -6~-4d, CTX 3.0 g -3~-2d, ATG 325 mg -4~ -1d used in 4 days. On November 30, the patient was transfused with 260 mL of HLA-matched unrelated donor (MUD 10/10) allogeneic peripheral blood stem cells, including 4.1×10^11^/L nucleated cells and 0.49% CD34+ cells. Because the patient weighed 57.5 kg, nucleated cells were transfused at 18.5 × 10^8^/kg, and CD34+ cells were transfused at 9.08 × 10^6^/kg. The patient was then treated with therapies to promote hematopoietic recovery, and methylprednisolone, cyclosporine A, and mycophenolate mofetil were given to prevent graft-versus-host disease (GVHD). The patient experienced persistent myelosuppression following donor cell infusion and developed a fever during the “agranulocytosis” stage, for which multiple anti-infective agents were administered. In December 2021 (30 days after transplantation), multiple nucleotide polymorphism (MNP) analysis of chimerism (using nest-generation sequencing) showed that the chimerism rate of the donor cells was 73.88%; bone marrow smear analysis showed that MCs accounted for 16.0% of cells, with MFC-MRD indicating that abnormal myeloblasts accounted for 0.38% and MCs accounted for 14.06%. Examinations at 37 and 51 days after transplantation showed that the chimerism rates were 91.87% and 68.81%, respectively; MFC-MRD was negative, but MC clones were present; and hemogram parameters had not improved/recovered. The patient still needed blood component transfusion; therefore, immunosuppressive therapy was reduced and stopped. Subsequently, due to the persistence of the disease, avapritinib tablets 200 mg po qd was started in January 2022. The chimera results were normal, but the hemogram parameters decreased more significantly. Thus, avapritinib treatment was adjusted to 100 mg po qd. Examinations in February showed that the chimera rate was 99.01%; the bone marrow smear analysis showed that MCs accounted for 4.8% of cells, with MFC-MRD indicating that MCs accounted for 3.66%. During treatment, the WBC count was 0.69-2.69×10^9^/L, N was 0.11-0.9×10^9^/L, HGB was 55–80 g/L, and the PLT count was 7-24×10^9^/L. The patient regularly received subcutaneous injections of recombinant human thrombopoietin and apheresis platelet transfusions. The dose of avapritinib tablets was adjusted to 75 mg po qd. Examinations on February 2022 showed that the chimera rate was 87.86%; the bone marrow smear analysis showed that MCs accounted for 5.0% of cells, with MFC-MRD showing that MCs accounted for 5.47%. On February 25, the patient was discharged from the hospital due to his strong wishes, and he continued to take avapritinib tablets orally after discharge.

In April 2022, MFC-MRD conducted at a local hospital showed that MCs accounted for 0.3% of cells. The results of a routine blood test on June 10 were as follows: WBC count, 2.18×10^9^/L; N, 0.7×10^9^/L; HGB, 101 g/L; and PLT count, 81×10^9^/L. The patient was in good condition, and blood transfusions were stopped. The patient continued to take avapritinib tablets orally.

**Figure 1:**
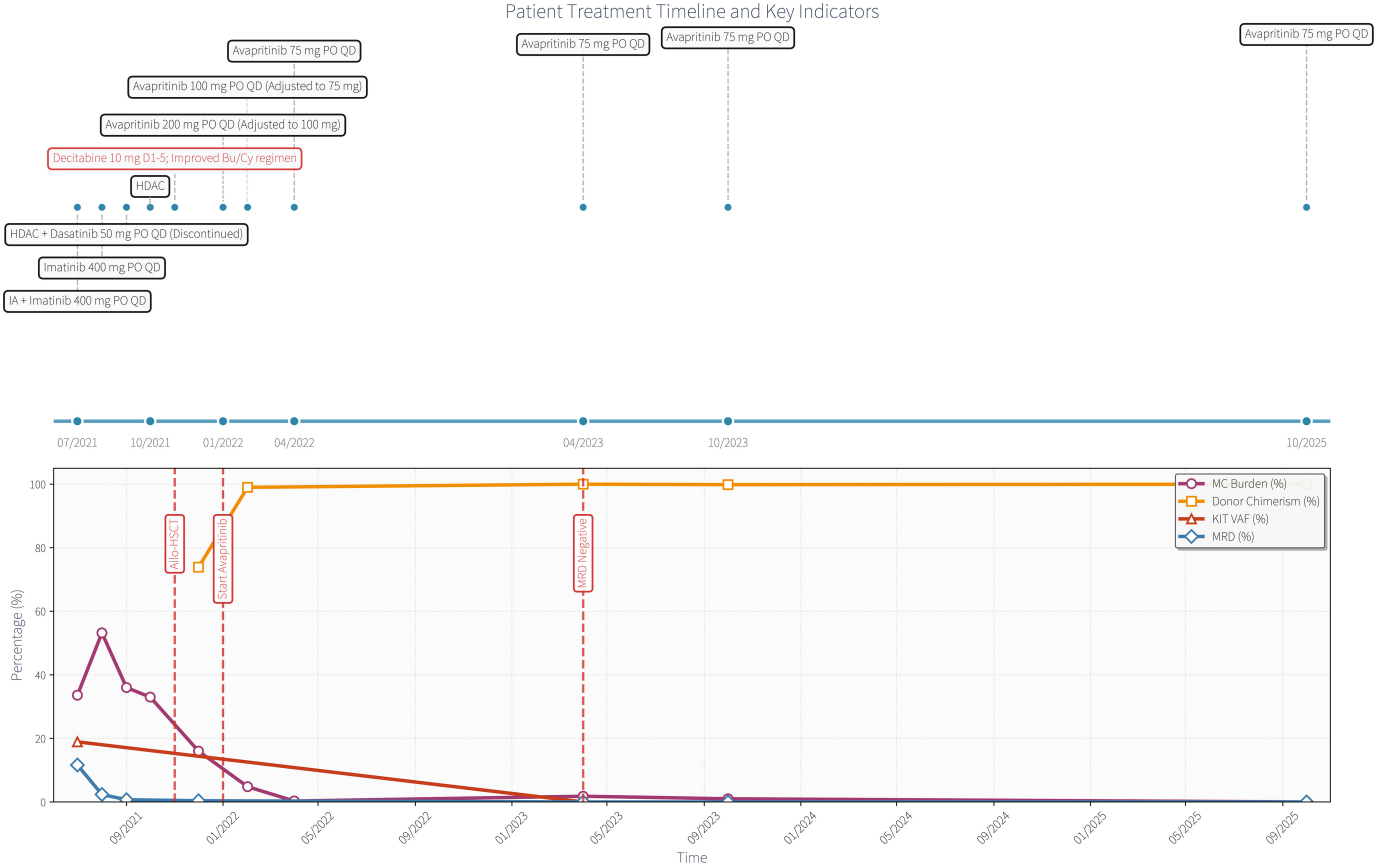
Patients treatment timeline and laboratory trends.

In April 2023 and October 2023, laboratory results confirmed complete remission (CR). Routine blood tests were normal, BM MCs were low (1.8% and 0%, respectively), with no dense aggregation on biopsy. MFC-MRD and *RUNX1::RNUX1T1* were negative. *KIT* gene mutation was negative. Donor chimerism was 100% and 99.85%, respectively (**Figure 1**). During the treatment period, myelosuppression was obvious at the beginning of the full dose. After the dose was reduced, the patient’s hemogram parameters gradually recovered, and no adverse reactions, such as bleeding or cognitive impairment, were observed. Long-term efficacy is still being investigated through follow-ups (**Figure 1**).

The patient achieved sustained CR with full donor chimerism and normalized hemogram parameters after the initiation of avapritinib, despite failed prior therapies. No severe adverse events were observed. The last follow-up was on October 25, 2025, with the patient remaining in CR and continuing maintenance therapy with oral avapritinib at 75 mg daily.

## Discussion and conclusion

According to the fifth edition of the World Health Organization (WHO) guidelines, SM has six subtypes, one of which is SM-AHN (4). SM-AHN, MC leukemia (MCL), and aggressive SM (ASM) are collectively called advanced systemic mastocytosis (AdvSM), and gene mutations in *TET2*, *SRSF2*, *CBL*, *ASXL1*, *RUNX1*, *EZH2*, and *RAS* can be detected in AdvSM (5). For the 3 high-risk mutations, the frequencies are 36-48% for *SRSF2*, 14-29% for *ASXL1*, and 13-23% for *RUNX1* (S/A/R), and these mutations are associated with poor OS (6). A recent retrospective study of 71 AdvSM patients undergoing allo-HSCT demonstrated that median OS varied significantly by subtype: 9.0 years for ASM/SM-AHN, 3.3 years for SM-AML, and 0.9 years for MCL ± AHN, with pre-transplant response to therapy and the absence of *KIT* D816V mutation significantly influencing outcomes (7). The therapeutic approach to SM-AHN involves determining whether the SM component, the AHN component, or both require priority treatment. Allo-HSCT may also be considered for the SM component, particularly when performed during an optimal response achieved with targeted agents such as midostaurin or avapritinib (1, 7).

*KIT* is a receptor tyrosine kinase and is involved in the proliferation of various types of cells, including MCs. *KIT* receptors on MCs are mainly activated by stem cell factor (SCF) and tightly regulate downstream signaling pathways to promote the proliferation and activation of normal MCs. *KIT* gene mutations lead to activation without ligands, resulting in increased transcription and decreased apoptosis in MCs, ultimately leading to abnormal MC proliferation and regulatory escape, thus causing SM (7, 8). There are two types of KIT-targeting tyrosine kinase inhibitors (TKIs). Type I TKIs (such as avapritinib) mainly bind to the active conformation of the *KIT* intracellular domain, and type II TKIs (such as imatinib) bind to the inactive conformation of the *KIT* intracellular domain. Because most SM patients carry the *KIT* D816V mutation and *KIT* is biased toward the active conformation, the efficacy of imatinib in these patients is poor (8, 9). In the patient reported in this case, none of the mutations detected in the *KIT* gene were D816V. Notably, preclinical studies have demonstrated that the avapritinib exhibits potent activity against various non-D816V *KIT* mutations and even wild-type *KIT*, supporting its potential applicability in such patients (10).

Many AdvSM patients undergo transplantation without achieving CR and remain at high risk of relapse. Avapritinib is a highly selective type I TKI for *KIT* D816V mutation, according to the clinical studies, the objective remission rate for the AdvSM subgroup with previous treatments was 71%, the rate of complete remission/complete remission with partial hematologic recovery was 19%, the median time to response was 2.3 months, which did not meet the median duration of remission, the 12- and 24-month OS was 80% and 65%, respectively, and the ORR for the SM-AHN subtype in this subgroup was 77%. The results showed that avapritinib could promote rapid, deep, and sustained remission. Further, the efficacy and safety of avapritinib were not affected by previous treatments. Avapritinib demonstrated definite efficacy in the treatment of *KIT* D816V-positive patients, but the efficacy in patients with *KIT* non-D816V mutation is unknown. In this report, a patient with a non-D816V *KIT* mutation who underwent Allo-HSCT exhibited persistent clonal MC proliferation and mixed chimerism. Treatment with avapritinib was associated with the achievement of 100% donor chimerism, a substantial reduction in MC burden, and clinical remission, without significant adverse reactions.

The clinical experience of applying avapritinib before transplantation is limited. Recently, a letter reported 3 patients with AdvSM treated with avapritinib and transplantation (bridging therapy), CR of SM was achieved in all patients, and the treatment was successfully changed to Allo-HSCT (11).

Experience concerning *KIT* inhibition after transplantation for the prevention or treatment of recurrence is also limited. This is only a case report. Prospective trials are needed to evaluate the effectiveness of Allo-HSCT combined with novel TKIs.

## Data Availability

The original contributions presented in the study are included in the article/supplementary material. Further inquiries can be directed to the corresponding author.

## References

[1] Laboratory Diagnosis Group Chinese Society of Hematology Chinese Medical Association the Mastocytosis Collaborative Network of China Depei W Suning C . [Chinese guidelines for diagnosis and treatment of systemic mastocytosis in adult patients (2022)]. Zhonghua Xue Ye Xue Za Zhi. (2022) 43:969–78. doi: 10.3760/cma.j.issn.0253-2727.2022.12.001, PMID: 36709101 PMC9939335

[2] Ustun C Reiter A Scott BL Nakamura R Damaj G Kreil S . Hematopoietic stem-cell transplantation for advanced systemic mastocytosis. J Clin Oncol. (2014) 32:3264–74. doi: 10.1200/jco.2014.55.2018, PMID: 25154823 PMC4876356

[3] DeAngelo DJ Quiery AT Radia D Drummond MW Gotlib J Robinson WA . Clinical activity in a phase 1 study of blu-285, a potent, highly-selective inhibitor of KIT D816V in advanced systemic mastocytosis (AdvSM). Blood. (2017) 130:2–2. doi: 10.1182/blood.V130.Suppl_1.2.2 28684445

[4] Khoury JD Solary E Abla O Akkari Y Alaggio R Apperley JF . The 5th edition of the world health organization classification of haematolymphoid tumours: myeloid and histiocytic/dendritic neoplasms. Leukemia. (2022) 36:1703–19. doi: 10.1038/s41375-022-01613-1, PMID: 35732831 PMC9252913

[5] Arock M Hoermann G Sotlar K Hermine O Sperr WR Hartmann K . Clinical impact and proposed application of molecular markers, genetic variants, and cytogenetic analysis in mast cell neoplasms: Status 2022. J Allergy Clin Immunol. (2022) 149:1855–65. doi: 10.1016/j.jaci.2022.04.004, PMID: 35430191

[6] Bibi S Langenfeld F Jeanningros S Brenet F Soucie E Hermine O . Molecular defects in mastocytosis: KIT and beyond KIT. Immunol Allergy Clinics North America. (2014) 34:239–62. doi: 10.1016/j.iac.2014.01.009, PMID: 24745672

[7] Lübke J Christen D Schwaab J Kaiser A Naumann N Shoumariyeh K . Allogeneic Hematopoietic Cell Transplantation in Advanced Systemic Mastocytosis: A retrospective analysis of the DRST and GREM registries. Leukemia. (2024) 38:810–21. doi: 10.1038/s41375-024-02186-x, PMID: 38448757 PMC10997505

[8] Ustun C Arock M Kluin-Nelemans HC Reiter A Sperr WR George T . Advanced systemic mastocytosis: from molecular and genetic progress to clinical practice. Haematologica. (2016) 101:1133–43. doi: 10.3324/haematol.2016.146563, PMID: 27694501

[9] Gilreath JA Tchertanov L Deininger MW . Novel approaches to treating advanced systemic mastocytosis. Clin Pharmacol: Adv Appl. (2019) 11:77–92. doi: 10.2147/cpaa.S206615, PMID: 31372066 PMC6630092

[10] Degenfeld-Schonburg L Gamperl S Stefanzl G Schruef AK Sadovnik I Bauer K . Antineoplastic efficacy profiles of avapritinib and nintedanib in KIT D816V(+) systemic mastocytosis: a preclinical study. Am J Cancer Res. (2023) 13:355–78., PMID: 36895976 PMC9989615

[11] Sriskandarajah P McLornan DP Oni C Wilson AJ Woodley C Ciesielska M . Advanced Systemic Mastocytosis with associated haematological neoplasm: Treatment with avapritinib can facilitate successful bridge to allogeneic haematopoietic cell transplant. Curr Res Trans Med. (2023) 71:103398. doi: 10.1016/j.retram.2023.103398, PMID: 37331225

